# Assessment of learners’ exposure to health education and promotion at school in the Limpopo Province of South Africa

**DOI:** 10.4102/phcfm.v8i2.932

**Published:** 2016-06-30

**Authors:** Oni. H. Tosin, Takalani G. Tshitangano

**Affiliations:** 1 Department of Public Health, University of Venda, South Africa

## Abstract

**Background:**

School participation and educational attainment among adolescents have been rising rapidly in the developing world. Thus, to attain Millennium Development Goal 6 (Combat HIV and/or AIDs, malaria and other diseases), it is crucial to seize the opportunity to educate and encourage teenagers about healthy choices and proper social behaviours that will continue into adulthood.

**Aim:**

This study aimed to assess the exposure of rural secondary school learners to health education and promotion at schools in the Limpopo Province of South Africa.

**Setting:**

This study was carried out at 10 secondary schools in Vhumbedzi educational circuit.

**Methodology:**

The study adopted a cross-sectional quantitative approach. Data were collected from 338 randomly selected learners from 10 secondary schools that make up a rural Vhumbedzi circuit in the Limpopo province. A self-administered questionnaire was used to collect data.

**Results:**

The findings showed that, 102 (66.07%) male and 121 (67.60%) female learners reported that they were taught about physical changes that occur during adolescence. In the same vein, most of the participants (*n* = 128, 84.39%) and (*n* = 152, 85.39%) males and females respectively claimed to have been taught about sexually transmitted diseases.

**Conclusion:**

In this study the secondary schools in the Limpopo Province of South Africa are making efforts to uphold and expose their learners to health education and promotion at school.

## Introduction

Since the 1950s, schools have been a focal setting for health promotion and health education with the aim of teaching young people about health and its determinants, so as to empower them to develop the skills to resist unhealthy lifestyles.^1^ During the 1990s, the World Health Organization, working jointly with the various organisations in each country, developed the health promoting schools initiatives, which involve a multi-factorial approach that covers teaching health knowledge and skills in the classroom, in order to change social and physical environment of the school, promote healthy development of school-going children and the communities in which they live and learn.^1^ The school health promotion initiatives were developed based on a medical model that seeks to prevent certain diseases or health problems and to address all the major public health problems that most adolescents are battling with in the 21st centuries, namely; drug and alcohol abuse, smoking, unhealthy eating, lack of physical activity, obesity, unwanted pregnancy, sexual transmitted infections and human immunodeficiency virus/acquired immunodeficiency syndrome (HIV and/or AIDS) as well as injuries among the adolescent population.^2,3,4,5^

Promoting the health and safety of adolescents as well as combating HIV and/or AIDs, malaria and other diseases – Millennium Development Goal (MDG) 6 – is of great importance for the future well-being of all nations. Adolescence represents a special period in the life cycle whereby an individual is no longer a child and not yet an adult. During this phase of life, young people tend to develop attitudes and most engage in health practices that affect their present safety and well-being, which may also influence their future either positively or negatively.^6,7,8^ Because school participation and educational attainment among adolescents have been rising rapidly in the developing world, it is therefore crucial to educate teenagers about healthy choices and proper social behaviours that will continue into their adulthood. In addition, efficacious transition of adolescents to adulthood, whether they are transitioning to marriage and parenthood, to household management, work, or to law abiding citizens, basically depends on the combination of good education and good health.^9^

However, educating and promoting adolescent well-being cannot be achieved by only one sector of the society. It takes the collective efforts of a broad range of societal sectors and institutions including; parents and families, health care providers, schools and tertiary institutions, community organisations and agencies that serve youth, faith-based organisations and adolescents themselves. Together, these bodies have a role to play in ensuring a nurturing structure and environment, as well as opportunities for growth that support and sustain the healthy development of young people.^12,13,14^

The question that comes to mind is ‘why should a country care about school health education and promotion?’ A country should care because the adolescents today are the leaders tomorrow. Thus, the health and well-being of our nation’s adolescents and youth are not matters of luck, chance, or random event. It must be a planned, well-designed, well-resourced, sustained, monitored, and evaluated program that should be incorporated into the nation’s schools. Moreover, a study of this nature is uncommon in South Africa. Whereas, if South Africa wants to achieve MDG 6, which aims to combat HIV and/or AIDs, malaria, and other diseases, a study of this nature cannot be undermined in this era. It is against this background that this study was undertaken.

### Purpose of the study

The aim of this article is to assess the learners’ exposure to health education and promotion at schools in the Limpopo Province of South Africa.

## Methodology

### Study design

Based on the purpose of the study, a quantitative cross-sectional descriptive survey design was adopted and this design is deemed suitable by the researchers because it describes and interprets phenomena that are in existence.^15^

### The study setting

The study was conducted at Vhumbedzi educational circuit situated in the east of Sibasa in the Vhembe District and north of Kruger National Park. Vhumbedzi circuit and secondary school learners were the phase one population of the study.

### Population and sample

The target population for this study was 10 secondary schools in the Vhumbedzi educational circuit with a total population of 5019 learners involved in the study.

### Sample selection and procedure

Based on the sampling frame of 5019, sample size of *n* = 370 was calculated using Slovin’s formula. A two-stage stratified sample selection process was used using grades and gender as strata within each of the 10 participating schools. Learners were selected randomly within each stratum based on population, which ensured proportional representativeness of grade and gender in the final sample (Table 1).

**TABLE 1:** Two-stage stratified sample selection process.

Name of school	Gender	Total population per grade and gender					Total	Proportional sample size per grade and gender					Total
	
		Gr 8	Gr 9	Gr 10	Gr 11	Gr 12		Gr 8	Gr 9	Gr 10	Gr 11	Gr 12	
Tondalushaka	M	94	36	83	54	63	330	7	2.7	6.1	4	4.6	24.3/25
F	78	56	75	44	60	313	5.8	4.1	5.5	3.2	4.4	23.1/23
**Total**	**172**	**92**	**158**	**98**	**123**	**643**	**12.8**	**6.8**	**11.6**	**7.2**	**9.0**	**47.4**
Ntsedzeni	M	34	57	81	63	19	254	2.5	4.2	6	4.6	1.4	18.7/19
F	38	49	63	79	44	273	2.8	3.6	4.6	5.8	3.2	20.0/21
**Total**	**72**	**106**	**144**	**142**	**63**	**527**	**5.3**	**7.8**	**10.6**	**10.4**	**4.6**	**38.7**
Vuvumutshena	M	47	29	30	23	18	147	3.5	2.2	2.2	1.7	1.3	10.9/11
F	54	25	43	37	11	170	4	1.8	3.2	2.7	0.8	12.5/13
**Total**	**101**	**54**	**73**	**60**	**29**	**317**	**7.5**	**4.0**	**5.4**	**4.4**	**2.1**	**23.4**
George Ntodeni	M	43	42	63	49	20	217	3.2	3.1	4.6	3.6	1.5	16/17
F	47	36	61	46	15	205	3.5	2.7	4.5	3.4	1.1	15.2/16
**Total**	**90**	**78**	**124**	**95**	**35**	**422**	**6.7**	**5.8**	**9.1**	**7.0**	**2.6**	**31.2**
Mpandeli	M	107	101	93	68	60	429	7.9	7.4	6.9	5	4.4	31.6/31
F	124	120	109	82	52	487	9.1	8.8	8	6	3.8	35.7/36
**Total**	**231**	**221**	**202**	**150**	**112**	**916**	**17**	**16.2**	**14.9**	**11**	**8.2**	**67.3**
Ranndogwana	M	65	45	88	52	26	276	4.8	3.3	6.5	3.8	1.9	20.3/21
F	55	67	75	52	14	263	4.1	4.9	5.5	3.8	1.0	19.3/20
**Total**	**120**	**112**	**163**	**104**	**40**	**539**	**8.9**	**8.2**	**12**	**7.6**	**2.9**	**39.6**
Funzwani	M	53	62	42	39	7	203	3.9	4.6	3.1	2.9	0.5	15/16
F	43	53	48	25	8	177	3.2	3.9	3.5	1.8	0.6	13/14
**Total**	**96**	**115**	**90**	**64**	**15**	**380**	**7.1**	**8.5**	**6.6**	**4.7**	**1.1**	**28**
Ntevhedzeni	M	15	14	30	11	13	83	1.1	1	2.2	0.8	1	6.1/6
F	11	4	30	22	10	77	0.8	0.3	2.2	1.6	0.7	5.6/7
**Total**	**26**	**18**	**60**	**33**	**23**	**160**	**1.9**	**1.3**	**4.4**	**2.4**	**1.7**	**11.7**
Limbedzi	M	26	22	26	23	22	119	1.9	1.6	1.9	1.7	1.6	8.7/10
F	26	18	42	24	11	121	1.9	1.3	3.1	1.8	0.8	8.9/9
**Total**	**52**	**40**	**68**	**47**	**33**	**240**	**3.8**	**2.9**	**5.0**	**3.5**	**2.4**	**17.6**
Milton Mpfumedzeni	M	110	86	120	73	56	445	8.1	6.3	8.8	5.4	4.1	32.7/32
F	93	88	102	90	57	430	6.9	6.5	7.5	6.6	4.2	31.7/33
**Total**	**203**	**174**	**222**	**163**	**113**	**875**	**15**	**12.8**	**16.3**	**12.0**	**8.3**	**64.4**

**Grand Total (*N* )**							**5019**	**Total Sample (*n*)**					**≈370**

*Source*: Project data analysis, 2012

### Data collection instrument

A self-administered questionnaire, adapted from the 2011 high school Youth Risk Behaviour Survey (YRBS) of the Centres for Disease Control and Prevention^16^ was used as an instrument in this study. The questionnaire was in English and required approximately 50–60 min to complete. Caution was taken to ensure that it was user-friendly and understandable.

### Instrument validity and reliability

To ensure validity and reliability, the instrument was adapted from the YRBS questionnaire of the Centres for Disease Control and Prevention^16^ to suit the local conditions. A wide range of literature was also consulted on the variables of interest. Also, the instrument was pre-tested on some volunteer learners in a school similar to the target population. Pre-testing results were used to rephrase and modify some aspects of the questionnaire thus making it suitable and comprehensible to the participants.

### Data collection process

The study was conducted over a 3-week period between October and November 2012. All 10 schools were visited by the research team to identify the learners who were to participate in the study. Dates for data collection were pre-arranged by circuit office and school authorities; and within each participating school, a special class was organised where the research team briefed the participants and assisted in facilitating the administration of the instrument and addressing issues arising thereof. The administration of the questionnaires lasted approximately 60 minutes.

### Data analysis

The Statistical Package for the Social Sciences (SPSS) version 21.0 software was used to analyse the data. Descriptive statistics were also used to summarise the data.

### Ethical considerations

An ethical clearance certificate (SHS/12/PH/03/0812) was obtained from the Research and Innovation Directorate of the University of Venda for the study. Further permissions were also acquired from the Department of Health – Limpopo Province, the Vhumbedzi circuit office, and each school administration. In addition, the participants and their parents signed an informed consent assuring anonymity, confidentiality, and voluntary participation before the administration of the questionnaire.

## Results

### Demographic profile of the participants

Though self-administered questionnaires were distributed to 370 learners proportionally according to grades, added together the response rate was 89% (*n* = 331). Thus, about 151 (45.6%) of the respondents were males whereas 54.4% (*n* = 180) of the respondents were females (Tables 2 and 3).

**TABLE 2:** Summary of the demographic profile of the respondents.

Gender	Frequency	%
Males	151	45.6
Females	180	54.4

*Source*: Project data analysis, 2012

**TABLE 3:** Summary of the frequency and percentages of learners; exposure to health education at schools.

Health education – Promotion	Male						Female					
	
	Yes		No		Unsure		Yes		No		Unsure	
					
	Number	Percentage	Number	Percentage	Number	Percentage	Number	Percentage	Number	Percentage	Number	Percentage
Physical changes that occur during adolescent	102	66.07	38	24.84	13	8.49	121	67.60	41	22.90	17	9.50
Menstruation	83	58.04	40	27.97	20	13.99	137	77.40	33	18.65	7	3.95
Wet dreams	98	64.05	43	28.10	12	7.85	87	51.48	54	31.95	28	16.57
Sexual harassment	113	74.83	31	20.53	7	4.64	127	70.56	38	21.11	15	8.33
How pregnancy occurs	121	80.13	23	15.23	7	4.64	146	81.11	20	11.11	14	7.78
Contraception	96	63.16	50	32.89	7	4.61	114	64.05	51	28.65	13	7.30
HIV/AIDS	144	94.74	5	3.29	3	1.97	169	94.95	7	3.93	2	1.12
Sexually Transmitted Infections	128	84.77	17	11.26	6	3.97	152	85.39	19	10.67	7	3.93
Nutrition	123	80.39	22	14.38	8	5.23	135	76.28	29	16.38	13	7.34
Where to get reproductive health services	92	60.93	44	29.14	15	9.93	103	57.54	46	25.70	30	16.76
TB	132	88.59	11	7.38	6	4.03	157	88.70	16	9.04	4	2.26
Personal hygiene and cleanliness	117	76.47	24	15.69	12	7.84	125	70.63	31	17.51	21	11.86
School environment hygiene and cleanliness	119	79.87	21	14.09	9	6.04	136	76.84	23	12.99	18	10.17
Chronic illnesses such as cancer, diabetes etc	124	81.58	22	14.47	6	3.95	141	79.21	25	14.05	12	6.74
Risk behaviours	121	79.61	25	16.45	6	3.94	153	85.00	22	12.22	5	2.78
Alcohol and drug abuse	139	92.05	8	5.30	4	2.65	162	90.50	16	8.94	1	0.56
Smoking	133	88.08	14	9.27	4	2.65	163	91.06	14	7.82	2	1.12
Your rights as adolescents	123	80.00	23	15.33	4	2.67	151	84.36	19	10.61	9	5.03
Importance of exercise	132	88.59	14	9.40	3	2.01	154	87.01	17	9.60	6	3.39
Dangers of obesity	116	77.85	22	14.77	11	7.38	129	71.67	38	21.11	13	7.22
Choice of termination of pregnancy	88	58.67	51	34.00	11	7.33	97	53.89	62	34.44	21	11.67
Danger of physical fights and violence	115	76.16	28	18.54	8	5.30	115	63.89	48	26.67	17	9.44

*Source*: Project data analysis, 2012

Responses above are based on whether learners have been taught the above during 2012.

### Health education and promotion at school

Of the respondents, 102 (66.07%) male and 121 (67.60%) female learners reported that they were taught physical changes that occur during adolescence (Table 1). In the same vein, most of the participants 128 (84.39%) and 152 (85.39%) male and female respectively claimed to have been taught about sexually transmitted diseases. Similarly, the majority of the learners 139 (92.05%) male and 162 (90.50%) female were taught about alcohol and drug abuse. Meanwhile, 144 (94.74%), and 169 (94.95%) male and female respondents were taught about HIV and/or AIDS. In addition, 115 (76.16%) males and 115 (63.89%) females attested to have being taught about the danger of physical fights and violence.

## Discussion

The findings of this study revealed that most of the learners had been taught various topics related to health education and promotion with 102 (66.07%) male and 121 (67.60%) female learners reporting being taught physical changes that occur during adolescence. In line with this, most of the respondents (*n* = 128, 84.39%) and (*n* = 152, 85.39%) male and female respectively claimed to have been taught about sexually transmitted diseases. Similarly, the majority of the learners (*n* = 139, 92.05%) male and (*n* = 162, 90.50%) female were taught about alcohol and drug abuse. Meanwhile, 144 (94.74%), 169 (94.95%) male and female respondents were taught about HIV/ and/or AIDS. Also, 115 (76.16%) males and 115 (63.89%) females attested to have been taught about the danger of physical fights and violence. It is not surprising that learners were exposed to these topics, because the South Africa government has sworn to ‘Put Children First’ giving their needs the highest priority.^3,5,10^ In order to uphold the rights of children and adolescents and make provision for them to attain their full potential in all facets of their lives, the Health, Education, and Social Development sectors were entrusted with a vital role in developing the National School Health Policy and Implementation Guidelines. This policy was developed and communicated to all schools.^5,11^ According to the policy, it was mandatory for all high schools to incorporate important health factors impacting on the development of children and youth of school-going age including issues relating to sexuality, HIV and/or AIDS and reproductive health, trauma and violence, substance abuse, and mental health problems. Such factors should be addressed through health promotion and health education activities and need to be incorporated into the life orientation area of the curriculum.^5,13^

Furthermore, 123 (80.39%) male and 135 (76.28%) female learners declared that they had been taught about nutrition. Of the respondents, 133 (88.08%) male and 163 (91.06%) female learners affirmed that they knew about the dangers associated with smoking. Because most of the respondents had learnt about risks associated with improper nutrition and smoking etc cetera; it is most likely that they will abstain from such acts, hence specific diseases and public health problems accompanying drug and alcohol abuse, smoking, unhealthy eating et cetera, can be minimised in the society.^3^ However, some students were unsure of whether or not they had been taught about particular issues; suggesting that they may have been taught about the subjects but could not recall this.

### Recommendations

In this study, a minor proportion of the participants claimed that they were not taught some of the topics; therefore it is very important to ensure that no student or learner is left out when it comes to exposure to health education and promotion, because knowledge is power and all the learners need to be empowered to make informed decisions. Also, more studies are needed in this area especially in other educational circuits of Limpopo Province.

### Limitations of the study

The fact that this study was conducted in one educational circuit limits the generalisation of the study findings to this circuit only.

## Conclusion

This study shows that secondary/high schools in the Limpopo Province of South Africa are making efforts to uphold and expose their learners to health education and promotion at school.
